# Molecular Cloning and Immunogenicity Evaluation of PpiC, GelE, and VS87_01105 Proteins of *Enterococcus faecalis* as Vaccine Candidates

**DOI:** 10.29252/.23.5.344

**Published:** 2019-09

**Authors:** Hamid Kazemian, Mohammad Reza Pourmand, Seyed Davar Siadat, Mehdi Mahdavi, Mohammad Hossein Yazdi, Peyman Avakh Majelan, Davoud Afshar, Mehdi Yaseri, Mehdi Davari, Muhammad Ibrahim Getso

**Affiliations:** 1 Department of Pathobiology, School of Public Health, Tehran University of Medical Sciences, Tehran, Iran;; 2 Department of Mycobacteriology and Pulmonary Research, Pasteur Institute of Iran, Tehran, Iran;; 3 Recombinant Vaccine Research Center, Tehran University of Medical Sciences, Tehran, Iran;; 4 Department of Pharmaceutical Biotechnology and Biotechnology Research Center, Faculty of Pharmacy, Tehran University of Medical Sciences, Tehran, Iran;; 5 Department of Microbiology and Virology, School of Medicine, Zanjan University of Medical Sciences, Zanjan, Iran;; 6 Department of Epidemiology and Biostatistics, School of Public Health, Tehran University of Medical Sciences, Tehran, Iran;; 7 Department of Medical Mycology, School of Public Health, Tehran University of Medical Sciences, International College, Tehran, Iran

**Keywords:** Enterococcus, Immunogenicity, Molecular cloning

## Abstract

**Background:**

Among the enterococci strains,* Enterococcus faecalis* is considered as one of the important nosocomial pathogens affecting immunocompromised patients. In this study, the immunogenicity of PpiC, GelE, and VS87_01105 proteins against enterococcal infection was investigated in a mice model.

**Methods:**

The genes encoding these proteins were cloned into pET21a expression vector, and the recombinant proteins were produced. Mice and rabbits were immunized with the purified recombinant proteins, and subsequently, mice were challenged with *E. faecalis *for the evaluation of their survival and bacterial clearances. The antibody responses to recombinant proteins were determined by ELISA assay, and *opsonophagocytic *activities of the antibodies were also measured. Passive immunization was performed using purified antibodies. Mice were challenged, and their survival and bacterial clearance were determined.

**Results:**

Immunized mice with PpiC, GelE, and VS87_01105 recombinant proteins showed 80%, 70%, and 40% survival rate, respectively. The survival rates among passively immunized mice that received 500 µg of IgG fraction in 100 µl PBS buffer of each of anti-PpiC, anti-GelE, and anti-VS87_01105 were 60%, 50%, and 20%, respectively. The rates of opsonization with anti-PpiC, anti-GelE, and anti-VS87_01105 antibodies at 1/10 dilution were 77%, 64%, and 23%, respectively.

**Conclusion:**

Based on our findings, PpiC, and GelE proteins can protect the mice against *E. faecalis* ATCC 29212 and effectively induce a protective antibody response. Thus, these proteins could be used as an additional therapeutic tool against enterococcal infections. Further studies to determine the role of PpiC in ligand binding and demonstration of epitope mapping may establish a credible target for vaccination.

## INTRODUCTION

Enterococci are considered as a part of the normal flora in humans and animals^[^^1^^]^. However, these bacteria may act as opportunistic pathogens in immunocompromised and hospitalized individuals^[^^2^^]^. In this condition, enterococci can cause a variety of clinical syndromes, including infective endocarditis^[^^3^^]^, bacteremia and sepsis^[^^4^^]^, surgical site infections^[^^5^^]^, neonatal meningitis^[^^6^^]^, cholecystitis^[^^7^^]^, peritonitis^[^^8^^]^, central nervous system infections^[^^9^^]^, wound infections^[^^10^^]^, and urinary tract infections^[^^11^^]^. *Enterococcus faecalis*, the most common isolate of nosocomial infections, accounts for more than 80-90% of human enterococcal infections^[^^12^^]^. 

The emergence of resistant enterococci appears to have accelerated in the past decade. *E. faecalis* has been shown to have potential to acquire resistance against some antimicrobial agents, especially vancomycin^[^^13^^]^. Meanwhile, resistance to the last-resort antibiotics, such as daptomycin and linezolid, is also emerging among enterococcus species^[^^14^^,^^15^^]^. This trend has raised a serious concern about the treatment of infected individuals. Moreover, horizontal gene transfer has been reported to play an essential role in the spread of resistant enterococci to other susceptible species^[^^16^^]^. Consequently, treatment of these infections is becoming increasingly challenging and might lead to increases in patient morbidity, mortality, and healthcare costs^[^^17^^]^. 

There is an urgent need to explore alternative strategies against enterococcal infections. Different surface antigens have been identified in *E. faecalis* that may be promising candidates for the development of vaccine against enterococcal infections. Only a few of these antigens have been tested in appropriate animal models^[^^2^^]^. In *E. faecalis, *PpiC homologue has been identified as a potential virulence factor. The protein confers resistance to high NaCl concentrations and ampicillin, and it is also involved in the folding and trafficking of extracellular proteins, especially penicillin binding proteins (PBPs)^[^^18^^]^. Gelatinase, encoded by *gelE* gene, is an extracellular metallo-endopeptidase that hydrolyzes collagen, gelatin, and small peptides. This protein is important for enterococcal virulence^[^^19^^]^. VS87_01105 is also a cell surface protein in *E. faecalis *that is being investigated in this study.

To the best of our knowledge, no appropriate prophylactic studies on these enterococcal proteins have been reported so far^[^^18^^]^. With the goal of introducing a new vaccine, the recombinant PpiC, GelE, and VS87_01105 were produced in *E. coli* host, and their immunogenic potentials were considered in a mouse model.

## MATERIALS AND METHODS

**Expression and purification of recombinant proteins**

DNA of *E. faecalis* ATCC 29212 was extracted using a DNA extraction kit (Qiagen, Germany) according to the manufacturer’s instructions. The extracted DNA was stored at -20 °C until further analysis. Amplification of *ppiC*, *gelE*, and *VS87_01105* genes was carried out using specific primers, shown in Table 1.

The amplified DNA fragments and pET21a expression vector (Novagen, USA) were digested with appropriate restriction enzymes (Fermentas, Germany). Ligations were carried out separately using T4 DNA ligase (Takara Shuzo Co., Japan) at 4 °C for 16 h. The ligation mixtures were transformed into *E. coli* DH5α competent cells by a standard CaCl_2/ _heat shock

transformation method. Bacterial colonies resistant to ampicillin were selected and confirmed by colony PCR using T7 primers. Recombinant vectors were extracted using the QIAprep spin miniprep kit (Qiagen, USA). Finally, the recombinant plasmids were confirmed by agarose gel electrophoresis and DNA sequence 

**Table 1:** Primers used in the study

**Primers **	**Oligonucleotides (5' → 3')** ^a^	**Restriction enzyme**	**Amplicon size (bp)**
*ppiC*-F *ppiC* -R	GCGCGCCATATGACGACCGCAACGAGTGATTC GCGCGCCTCGAGTTTTTTTGCTTCTTGAAGAATATTGATTTTT	*Nde*I *Xho*I	750
			
*gelE*-F *gelE* -R	GCGCGCCATATGGCAGAAGAACAAGTTTATTCAGAAA CTCGAG	*Nde*I *Xho*I	1530
			
			
*VS87_01105* -F *VS87_01105* -R	GCGCGCCATATGGCAGAAGAACAAGTTTATTCAGAAA CTCGAG	*Nde*I *Xho*I	1887

analysis. Expression of recombinant proteins in E. coli BL21 (DE3) and purification of the recombinant proteins were performed as described before[20].

a Artificial restriction sites are underlined.

**Western-blot analysis**

Western-blot analysis was performed to confirm the successful protein expression using His-tag monoclonal antibody conjugated to horseradish peroxidase (HRP; Thermo Fisher Scientific, Lithuania). The recombinant proteins were separated on a 12.5% polyacrylamide SDS gel. The protein bands were then transferred onto PVDF membrane using a semi-dry blotting system (Bio-Rad, Hercules, CA, USA) at 4 °C for 90 min. Membranes were blocked by incubation in PBS containing 3% skimmed milk and 0.05% Tween 20 at 4 °C overnight. After blocking, membranes were washed three times with PBS containing 0.05% Tween 20 and then incubated with a 1:1000 dilution of anti-His Tag HRP-conjugated monoclonal antibody at 25 °C for 1 h. Finally, the membranes were washed three times with PBS 1 containing 0.05% Tween 20 and exposed to 3,3-diaminobenzidine solution (Sigma-Aldrich, USA) until the appearance of bands. 

**Mice immunizations**

This study was conducted using 6–8-week-old BALB/c mice (Ethical number: IR.TUMS.SPH.REC. 1396.2067). The mice were obtained from Pasteur Institute of Iran (Karaj, Iran) and kept in cages in an animal house facility. Experiments were performed in accordance with animal protocols approved by the Institutional Animal Care and Use Committee of Tehran University of Medical Sciences. Complete Freund's adjuvant was used for initial injections and incomplete Freund's adjuvant for subsequent boosts. Mice were divided into four groups with 16 mice in each group, namely PpiC + adjuvant, GelE + adjuvant, VS87_01105+ adjuvant, and PBS. The three first groups were immunized with adjuvant + 30 µg of the corresponding protein. Also, negative control group of mice was injected with PBS buffer. Entirely, all mice were subcutaneously immunized every 14 days to a total of three doses. To check antibody titers, before each immunization, blood samples were obtained from mice by tail bleeding. 

**Rabbit immunizations**

White New Zealand male rabbits were purchased from Pasteur Institute of Iran. They weighed between 2-3 kg. All the rabbits were immunized by multi-point injection in the back with adjuvant along with 50 µg of recombinant proteins, 3 times at 14-day intervals. For ELISA identification, blood samples were taken from the marginal vein of the ear after seven days of each immunization. Seven days after the final boost, all immunized rabbits were exsanguinated by heart puncture, and the serum was separated from blood cells by storing at 37 °C for 1 hr and centrifugation at 3000 g at room temperature for 15 min. Antibodies were purified using protein A affinity column chromatography (Bio-Rad Laboratories, Richmond, California, USA). The purified antibodies were dispensed into suitable containers and quantified with Bradford and stored at -20 °C.

**Enzyme-linked immunosorbent assay (ELISA)**

The ELISA assay was performed after last immunization to determine the specific total IgG antibody, IgG_1_, and IgG_2_a isotypes. The recombinant proteins (10 μg/ml in PBS) were coated into 96-well polystyrene plates (Greiner Bio-One, Frickenhausen, Germany) by overnight incubation at 4 °C. The wells were washed three times with PBS Tween 20 and then blocked with PBS containing 4% skimmed milk at RT for 1 h. The plates were then washed three times using PBS-T20, and diluted sera were added into the wells at the dilutions of 1:100 to 1:12800 and incubated at RT for 1 h. Following rinses, peroxidase-conjugated goat anti-mouse IgG (Cyto Matin Gene, Isfahan, Iran; diluted 1:2000) was added and incubated at room temperature for one hour. Finally, tetra-methyl benzidine (TMB) substrate solution (Cyto Matin Gene) was added, and the reaction was stopped with adding 100 µl of 2N H_2_SO_4_.

**Cytokine assay**

For cytokine ELISA assay, blood samples were obtained from mice by retro-orbital bleeding after 16 h of the last immunization. Sera were separated from blood cells by storing at 37 °C for one hour and centrifugation at 12000 g at room temperature for 10 min. The samples were analyzed for IL-4, IL-17, and IFN-γ using a sandwich ELISA assay kit (Mabtech, Sweden) according to the manufacturer’s recommendations.

**Opsonophagocytic assay**

The opsonophagocytic assay was performed according to the method of Ames *et al.*^[21]^. Briefly, bacterial suspensions were prepared at the concentration of 1 × 10^8^ CFU/mL in 1% bovine serum albumin (BSA). A final concentration of 2 × 10^7^ mL mouse macrophages in RPMI-1640 supplemented with 10% heat-inactivated fetal bovine serum was used. Besides, baby rabbit sera (Pasture Institute of Iran) were used as a complement source, and a dilution of pooled pre-challenge anti-r-PpiC, anti-r-GelE, and anti- r-VS87_01105 from each group was used. Complement of antisera was inactivated by heating at 56 °C for 30 min. For the opsonophagocytic assay, the bacteria were incubated with an equal 1:10 volume of heat-inactivated polyclonal IgG at 22 °C for 1 h. To eliminate excessive antibodies, wells were washed twice with BSA (1% [w/v]). Then 100 μL of mouse macrophage was mixed with 100 μL of complement in a sterile 96-well plate (Greiner Bio-One) and incubated in a shaker at 37 °C for 90 min. Finally, 25 μL of the mixture was removed, diluted in saline and plated for bacterial enumeration. The opsonic activity of immune sera was compared to non-immune normal rabbit serum (NRS), statistically. This experiment was performed in triplicate for each quantity. The following formula was used for calculation of the percentage of killed bacteria.

**Fig. 1:**
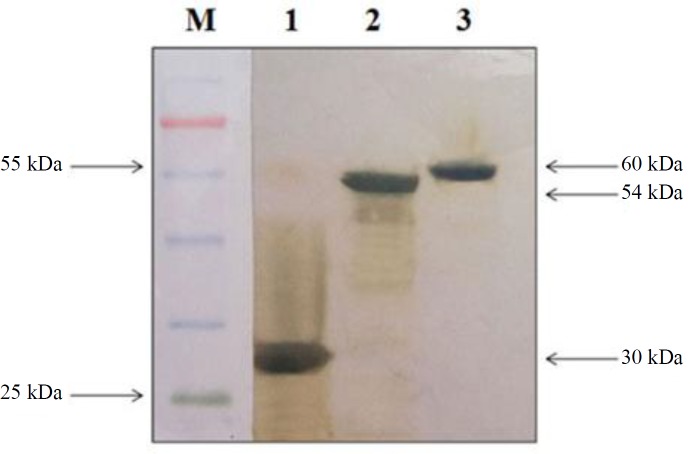
Western-blot analysis of recombinant proteins. Lane 1, purified PpiC; lane 2, GelE (2); lane 3, VS87_0110; M, protein size marker

Opsonophagocytosis (%) = (1 - [CFU of immune serum/CFU of pre-immune serum]) × 100

**Experimental challenge**

A mouse bacteremia model was induced in the immunized mice and control groups with *E. faecalis* ATCC 29212 (1 × 10^8 ^CFU). Bacterial inoculum was injected via the tail vein (intra-venus [i.v.]). Twenty-four hours after challenge, the blood collection was performed from the tail vein of the mice and diluted serially into PBS and were then cultured on blood agar plates. This procedure was followed until seven days after infection. Moreover, mice were observed daily for mortality until 14 days after infection. 

**Passive protection assays**

To explore the protectivity of rabbit IgG antibodies fraction against PpiC, VS87_01105, and GelE, 10 female BALB/c mice were employed in each experimental group. The dose of 500 µg of IgG fraction in 100 µl of PBS buffer was used for each antibody. Then equal volumes of bacterial suspension (approximately 1 × 10^8 ^CFU) and 500 µg of IgG antibody fraction were administered i.v. to 10 mice in each group. The CFU was counted out at 1, 2, 3, and 7 day(s) after bacterial challenge. The survival time of each mouse was monitored daily for 21 days.

**Statistical analyses**

The Kruskal-Wallis test was used for the statistical analysis of the data. The groups with significant differences were further analyzed by post hoc tests. The limit of statistical significance was a *p* value of 0.05. All data analyses were performed using the Statistical Packages for Social Sciences (SPSS) version 22.

## RESULTS

**Purification and characterization of recombinant proteins**

**Fig. 2:**
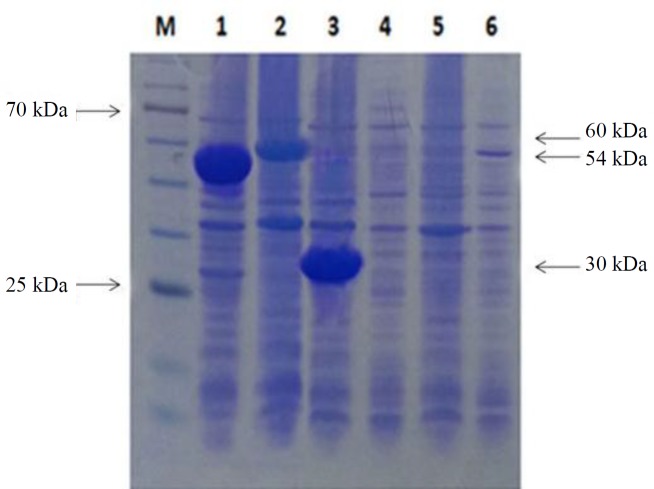
Expression of PpiC, GelE, and VS87_01105 proteins in 12.5% gel agarose. Lanes 1, 2, and 3, GelE, VS87_01105, and PpiC proteins after induction, respectively; lanes 3, 4, and 5, GelE, VS87_01105, and PpiC proteins before induction, respectively. M, protein size marker

All three recombinant proteins were expressed as inclusion body in *E. coli* expression host BL21 (DE3). The recombinant proteins were then purified by Ni-NTA chromatography. All the recombinant proteins were of predicted size based on the migration on SDS-PAGE gels (approximately 60 kDa for GelE, 55 kDa for VS87_01105, and 30 kDa for PpiC). Western-blot analysis using anti-His monoclonal antibody revealed positive bands with the correct size of recombinant proteins (Figs. 1 and 2).

**Mice immune response to antigens**

**Fig. 3:**
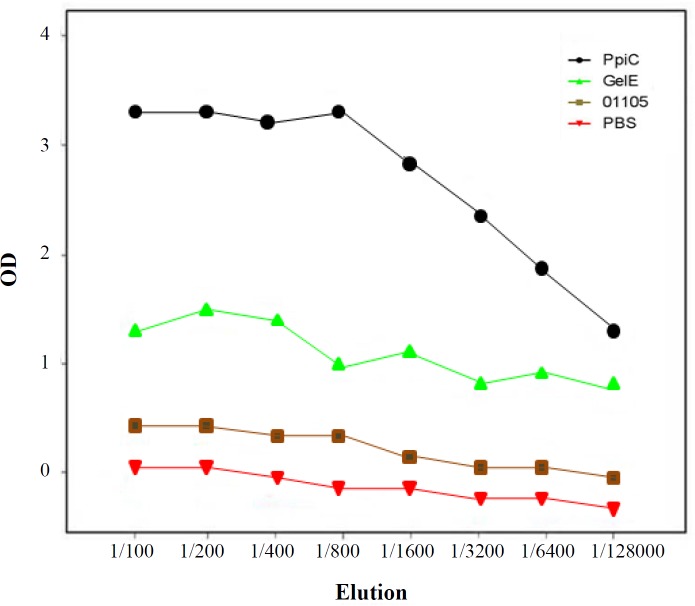
Titration of specific antibodies in sera of immunized mice. Two weeks after the last immunization, serum samples of the mice were collected and pooled. The sera were serially diluted and were coated on recombinant proteins. Different groups of mice each consisting of 16 mice were administrated with either the recombinant antigens PpiC, GelE, and VS87_01105. Mice injected with PBS alone served as controls. The titers of specific antibodies were significantly high in mice (p < 0.05

**Fig. 4:**
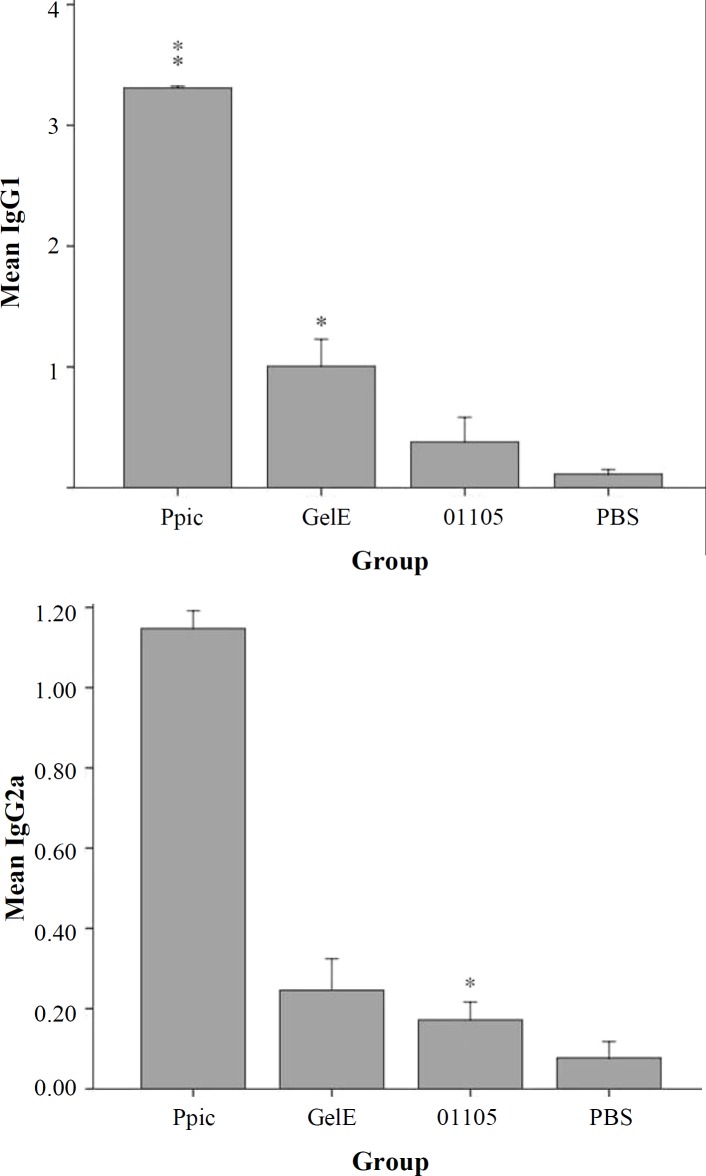
Immunoglobulin isotyping assay in sera of immunized mice. Two weeks after the last immunization, serum samples of the mice were collected and pooled. The sera were serially diluted and were coated on recombinant proteins. Different groups of mice each consisting of 16 mice were administrated with either the recombinant antigens PpiC, GelE, and VS87_01105. Mice injected with PBS alone served as controls. The IgG1 levels were higher than IgG2a levels in comparison to the control groups. The IgGs levels against PpiC were higher than other proteins. *p < 0.05 and **p < 0.01

Experimental sera collected from the mice after the last booster injection were assessed in all groups of mice by indirect-ELISA. Data analysis showed that antibody titers PpiC and GelE recombinant proteins induced a significant antibody response in mice when compared with the PBS. VS87_01105 protein, however, did not induce any significant antibody response in the mice model (*p* < 0.05; Fig. 3). The types of immune responses to PpiC, VS87_01105 and GelE antigens were further examined by measuring the levels of IgG_1_ and IgG_2_a isotypes. The IgG_1_ levels were higher than IgG_2_a levels in comparison to the control groups with statistical significance (*p* < 0.05; Fig. 4).

**Survival rate and bacterial clearance in immunized mice**

**Fig. 5:**
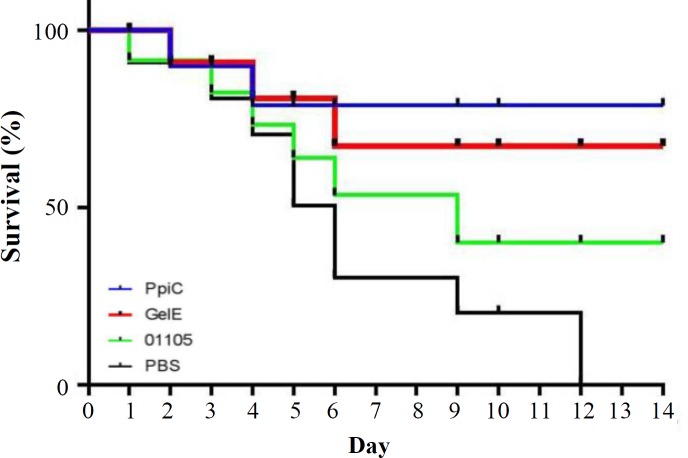
Cumulative survival rates of mice challenged with the serotype E. faecalis ATCC 29212. The mice were intraperitoneally infected with 1 × 108 CFU of E. faecalis. Survival rate of each group was checked for 14 days. There were statistically significant differences (p < 0.05) between the immunized mice and control groups

**Fig. 6:**
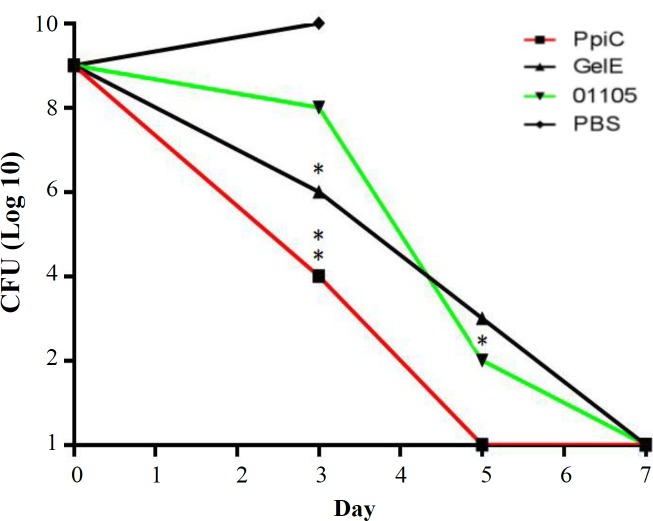
Clearance rate of mice from enterococcal bacteremia. The mice were intraperitoneally infected with 1 × 108 CFU of E. faecalis ATCC 29212. The CFUs in the blood were determined 3, 5, and 7 days after challenge. There were statistically significant differences (p < 0.05) between the immunized mice and control groups. *p < 0.05 and **p < 0.01

To evaluate the protection elicited by immunization with recombinant PpiC, GelE, and VS87_01105 proteins, BALB/c mice were immunized with these proteins (formulated in adjuvant) and then challenged with *E. faecalis* ATCC 29212. The mice immunized with the recombinant PpiC showed a statistically significant improvement (*p* < 0.05) in survival rate after bacterial challenge (Fig. 5). The median survival times for mice immunized with the experimental antigens were significantly longer than the times used for mice that received PBS alone (*p* < 0.05). The highest survival rate was observed in mice that received PpiC antigen. Administration of VS87_01105 antigen only delayed the time to death but failed to protect mice from death. The clearance rates from enterococcal bacteremia following immunization with PpiC, GelE, and VS87_01105 recombinant proteins were within 96 h after the challenge, although mice immunized with PpiC have been cleared at 48 h after infection (Fig. 6). The comparison of the immunized groups with the control group showed a significant difference at 24 h (*p* < 0.05), but no significant differences were seen among the immunized groups (*p* > 0.05).

**Cytokine assay**

**Fig. 7:**
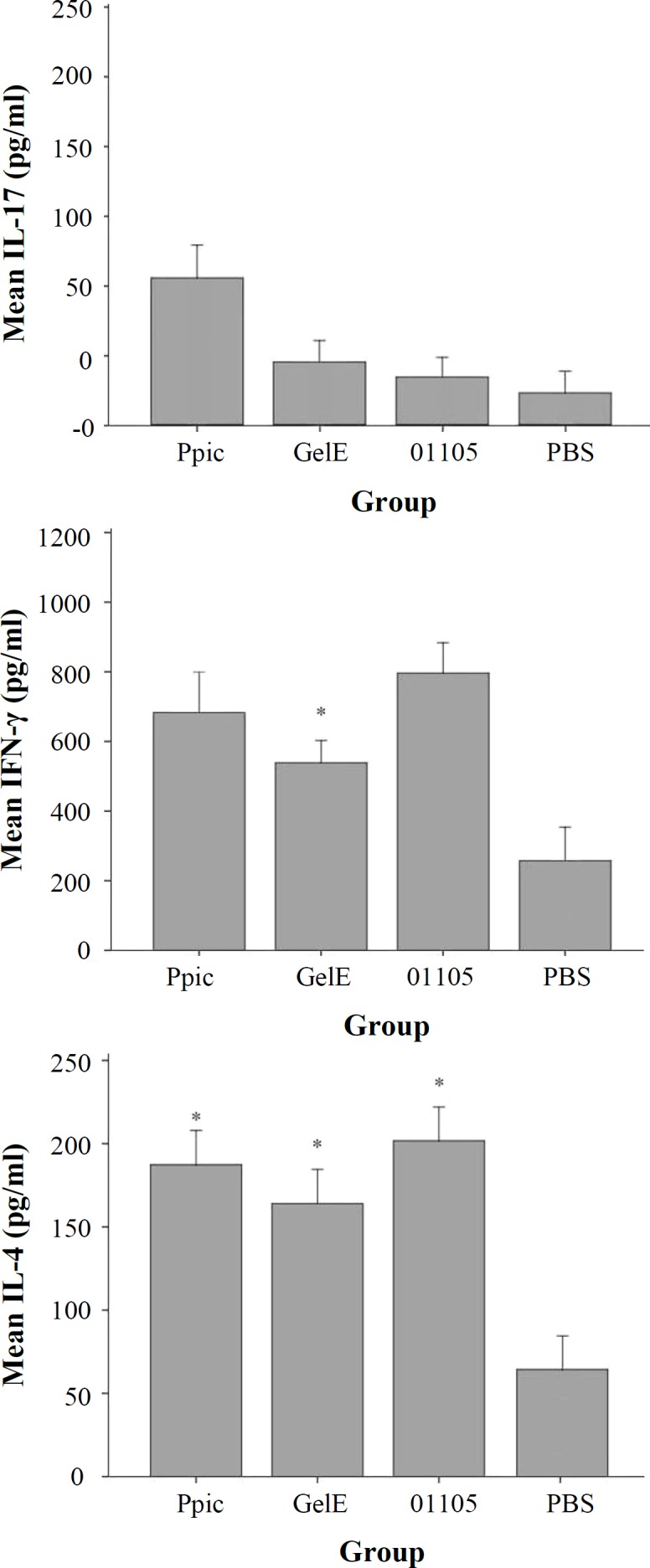
Levels of IL-4, IL-17, and IFN-γ cytokines (pg/ml) in mice groups. Control groups were not stimulated by antigens. The level of IL-17 was very low compared to the two other cytokines and did not show statistical differences with mice group that received PBS (p > 0.05). There were not statistically significant differences (p > 0.05) between the groups (*p < 0.05

A significant amount of IFN-γ and IL-4 cytokines was produced in immunized mice, whereas the IL-17 cytokine level was very low (Fig. 7). There were not statistically significant differences (*p* < 0.05) between the groups. The level of IL-17 in the mice immunized with PpiC vaccine shows a significant difference (*p* < 0.05) in comparison to other vaccinated and control groups.

**Opsonophagocytic activity**

**Fig. 8:**
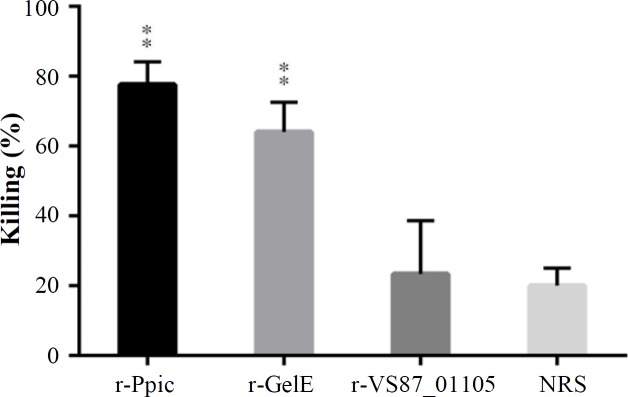
The opsonic activity of PpiC, GelE, and VS87_01105 proteins. (A) At a dilution of 1:10, the anti-r-PpiC and anti-r-GelE sera induced a higher significant increase in the opsonophagocytic responses than the VS87_01105 sera groups against the E. faecalis ATCC 29212, two weeks after the last dose (p < 0.05). A statistically significant difference was observed among the anti r-PpiC, anti- GelE sera, and NRS. There is no significant difference between anti-VS87_01105 and NRS (p ˃ 0.05). **p < 0.01

The bioactivity of antibodies against recombinant proteins to promote phagocytosis of bacteria was evaluated by incubating *E. faecalis *with pooled and diluted anti-r-PpiC, anti-r-GelE, and anti-r-VS87_01105 antibodies (pre-challenge serum) and mouse macrophages in the presence of rabbit complement. The anti-r-PpiC and anti-r-GelE sera induced a higher significant increase in the opsonophagocytic responses (77% and 64%, respectively) at a dilution of 1:10 than the VS87_01105 (23%) sera groups against the *E. faecalis* ATCC 29212, two weeks after the last dose (*p* < 0.05). A statistically significant difference (*p *< 0.01) was observed in respiratory burst among the anti-r-PpiC and anti-r-GelE sera and NRS (<1%), as shown in Figure 8. There was no significant difference between anti- VS87_01105 and NRS (*p* ˃ 0.05).

**Passive protection and enterococcal challenge**

**Fig. 9:**
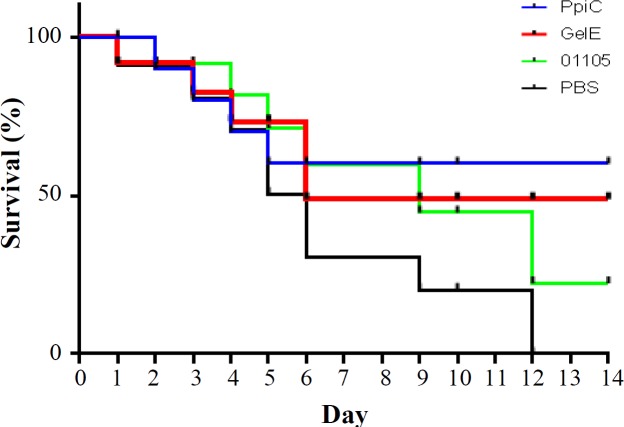
The Kaplan-Meier curve (cumulative survival) of BALB/c mice challenged with E. faecalis. The cumulative survival rate was plotted against time. BALB/c mice immunized with the recombinant PpiC showed a statistically significant improvement (p < 0.05) in survival rate after bacterial challenge with E. faecalis ATCC 29212 compared to other mice groups

**Fig. 10:**
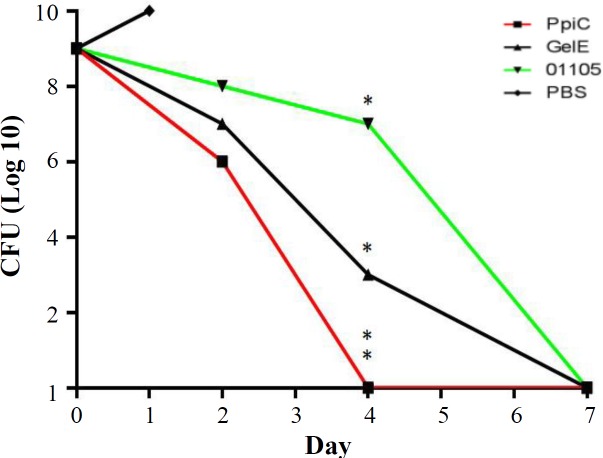
Enterococcal clearance of mice receiving polyclonal antibodies. The clearance rates from enterococcal bacteremia also indicated that immunization with PpiC, GelE, and VS87_01105 recombinant proteins resulted in bacterial clearance within seven days after the challenge, although mice immunized with PpiC were cleared at 96 h after infection. The comparison of immunized groups with each other and control groups showed a significant difference at 96 h with control groups (p < 0.05), while no significant differences were seen among the immunized groups (p > 0.05). *p < 0.05 and **p < 0.01

To examine the protectivity of rabbit-specific IgG antibodies against enterococcal infection, mice were passively immunized with rabbit hyper-immune IgG fraction before bacterial challenge. Mice that received hyper-immune sera lived significantly longer than those received sera from non-immunized rabbits (*p* < 0.05). The survival rates of the mice groups that received anti-PpiC, anti-GelE, and anti-VS87_01105 antibodies were 75%, 50% and 25%, respectively (Figs. 9 and 10).

## DISCUSSION

*E. faecalis* is considered as an important nosocomial pathogen in individuals whose immune systems are compromised^[^^22^^]^. Enterococcal infections result from the capacity of the bacteria to adhere, colonize and invade onto host tissues and to produce extracellular enzymes and toxins as well as various virulence factors that enhance the severity of the infection^[^^23^^]^. Enterococcus is a problematic pathogen that is becoming increasingly hard to treat even with available antibiotics. The identification of protective antigens is needed to facilitate vaccine-based prophylactic approaches. Previous studies have demonstrated that surface-exposed proteins, closely related to peptidoglycan, can be targeted as potential candidates for vaccine production against enterococcal pathogens^[^^18^^,^^24^^]^.

*E. faecalis* has many surface proteins on its cell wall that play major physiological and structural roles. By *in silico* approaches, many surface-exposed proteins have been characterized to be promising vaccine candidates in different species of bacteria^[^^25^^-^^27^^]^. In this study, the immunogenicity and protectivity of PpiC, GelE, and VS87_01105 recombinant proteins on *E. faecalis* were investigated.

Our results showed that the PpiC protein elicited protective antibodies in mice challenged with *E. faecalis* ATCC 29212. A similar study has demonstrated that cell surface proteins, including PipC could produce specific, opsonic and protective antibodies against enterococcal infections and thus can be considered as vaccine candidates^[^^18^^]^. Our results demonstrated immunogenic capacities of PpiC, GelE, and VS87_01105 recombinant proteins. Passiveimmunization with the antibodies raised against different proteins promoted the clearance of *E. faecalis* ATCC 29212 in the immunized mice in comparison with the normal mice sera. These results are comparable to the protection achieved by antibodies raised against the previously reported antigens, such as SagA and LTA^[^^28^^,^^29^^]^. The high clearance activity of PpiC in comparison to the two other proteins may be associated with the high immunogenicity and possible surface-exposing features in this protein, although this area requires further investigation. Similarly, the high survival rate of mice immunized with PpiC protein has formerly been reported^[^^18^^]^. In this study, recombinant proteins and PBS were emulsified with Freund’s complete adjuvant (first vaccination) and Freund’s incomplete adjuvant (boosts), which generated high titers of anti-PpiC and anti-GelE. This result is comparable to other study that used this adjuvant^[^^30^^]^.

In our study, the plasma level of IFN-γ produced in mice immunized with PpiC, GelE, and VS87_01105 recombinant proteins was higher than that of IL-4 and IL-17. The high level production of this cytokine in response to recombinant proteins might be due to the stimulation of Th-1 lymphocytes. A number of studies have reported that certain cytokines and other components of innate immune systems are essential for bacterial clearance in animal models^[^^31^^,^^32^^]^. However, the plasma level of IFN-γ raised only within six hour post enterococcal challenge^[^^33^^]^. Suppression of innate immune system by *E. faecalis* has been reported to be associated with the blockade of NF-KB signaling by reducing microbial clearance in the model mouse^[^^24^^]^. antibodies in bacterial opsonization, need to be further tested^[^^29^^,^^35^^]^.

In a previous study that conducted by Leendertse *et al.*^[^^34^^]^ certain enterococcal protein has elicited specific opsonic antibodies that could mediate opsophogocytic killing in immunized animal models. In our study, we demonstrated that PpiC and GelE proteins induced a significant increase in the opsonophagocytic responses (77% and 64%, respectively) against the *E. faecalis* ATCC 29212. The opsonophagocytic activity of the sera was found to be dose-dependent, with maximum activity at 1:10 dilution. A similar pattern of activity has also been reported in a previous study^[^^34^^]^. Although the recombinant proteins induce high levels of antibodies, antibodies against VS87_01105 protein have been shown to have a low level of opsonic activity. We suppose that this protein may not be adequately exposed to the antibodies. This insufficient exposure may be due to difference in opsonic capacity of antibody subtypes. Other antibody subtypes such as IgM antibodies, which are more effective than IgG The protection offered by passive immunization indicates that the defense against entrococcal infections obtained from a balanced Th1 and Th2 stimulatory response owing to significant plasma level of INF-γ and IL-4 in the immunized models. The effectiveness of passive protection against enterococcal infections using sera from mice immunized with other surface proteins has also been reported^[^^18^^]^. Such findings reveal that these surface-related proteins can be promising vaccine candidates for enterococcal infections.

Results of the present research clearly demonstrate that the PpiC recombinant protein efficiently stimulate cell-mediated immunity and results in significant opsonophagocytotic activity against *E. faecalis* ATCC 29212 infection. Our results predict that this enterococcal polysaccharide protein has the potential for broad protective coverage against *E. faecalis infection. *Both PpiC and GelE proteins can be used as candidates for vaccine development, especially in combined form for better efficacy. Further studies to determine the role of PpiC in ligand binding and demonstrate the epitope mapping may qualify the protein as a credible target for vaccination.
